# Non-Thrombotic Pulmonary Embolism

**DOI:** 10.5334/jbr-btr.1226

**Published:** 2016-11-19

**Authors:** Benoît Ghaye

**Affiliations:** 1 Cliniques Universitaires St Luc, Université Catholique de Louvain, BE

**Keywords:** Pulmonary artery, Embolism, Non-thrombotic embolism, Tumoral embolism, Fat embolism

The microvascular pulmonary bed has the property to filter any tissue or substance, including among others fat, air, tumor, septic material, organ fragments and foreign material, of approximately 10 µm or greater that gains access to the venous systemic circulation. This can result in obstruction to blood flow, injury to the lung parenchyma and impairment of gas exchanges.

## Tumor Embolism

**Figure 1:**
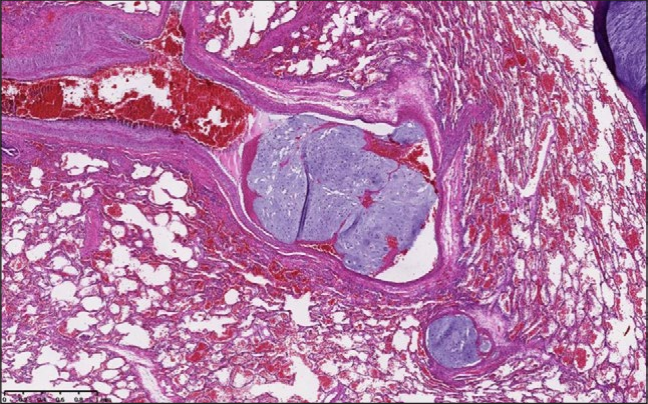
Microscopic view of tumor embolism. Fragments of chondrosarcoma are demonstrated inside pulmonary arteries.

**Figure 2:**
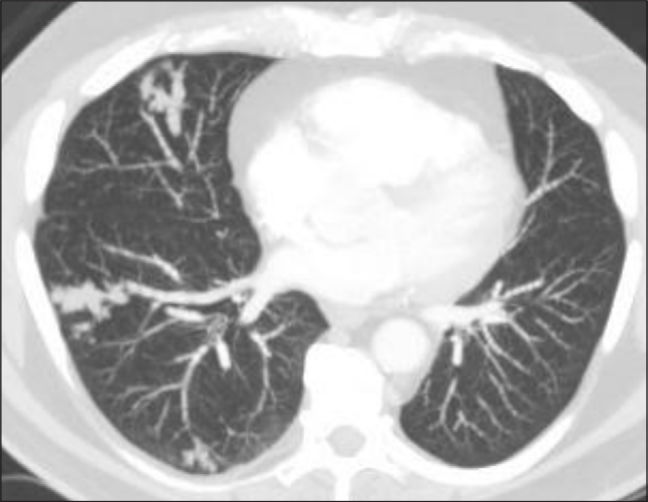
Tumor embolism in a patient with a past history of colic adenocarcinoma. Three foci of intra-arterial metastatic tumor proliferation are seen in the right lung showing beaded arteries and tree-in-bud patterns.

Tumor embolism is not rare at autopsy (3–26%), particularly in solid tumors that tend to invade systemic veins (hepatocellular, renal cell, breast, chondrosarcoma or lung carcinoma) (Figure 1). The condition remains however underdiagnosed in clinical routine, even on computed tomography (CT) or magnetic resonance (MR). Most tumor emboli are microscopic and involve peripheral pulmonary arteries (PAs) and arterioles, except the right atrial myxoma, renal cell carcinoma or chondrosarcoma that may embolize to the central and segmental PAs. Symptoms include progressive dyspnea and subacute pulmonary hypertension or a more acute presentation that may simulate pulmonary embolism (PE). On CT, they may present as multifocal dilations or beading of PAs, sometimes showing a tree-in-bud pattern (Figure 2). Areas of mosaic perfusion, pulmonary infarction or even a pattern of pseudo-consolidation of lung parenchyma have been reported. Findings that should allow differentiation from cruoric PE include absence of risk factor for PE, absence of regression under anticoagulation, lobulated or heterogeneous components, enhancement after CM administration and transmural extension. They should also be differentiated from filling-in of dilated bronchial structures, i.e., mucous plugging in bronchiectasis or endobronchial growth of tumor.

## Septic Embolism

Embolism of septic material in the PAs may be secondary to right heart endocarditis, infected central venous line or pacemaker, septic phlebitis, Lemierre syndrome, hydatid disease and osteomyelitis. The risk is particularly higher in immunocompromised patients. On imaging, pulmonary septic embolism may result in pulmonary nodules showing a vascular distribution and often central necrosis or cavitation. The halo sign may be associated and suggests haemorrhage or a pulmonary infarct. Mycotic pseudoaneurysm may occasionally be seen in the wall of cavitary nodules and be responsible for hemoptysis. Most of them will resolve under antibiotic therapy. Occasionally, septic emboli may present as clot in more central PAs.

## Fat Embolism

Fat embolism syndrome (FES) is extensively debated in the literature, concerning etiology, risk, pathophysiology, diagnosis and treatment. FES is a phenomenon secondary to fat embolism. The causes of FES are multiple and include traumatic, surgical, and nontraumatic origins. The classic triad consists of respiratory distress, cerebral dysfunction and a petechial rash. The first step of fat embolism is a traumatic mechanical release, due to a local high pressure, of fat globules from the bone marrow (or adipose tissue) into small veins, which eventually embolize. An initial mechanical effect that is severe enough can lead to right heart strain or failure. Otherwise pulmonary findings including hypoxemia, dyspnea and tachypnea are usually the earliest symptoms. Eighty percent of circulating fat globules are trapped in the pulmonary vascular bed while a minority will enter the systemic circulation and be responsible for the systemic symptoms. Degradation of fat by an endothelial lipase produces free fatty acids, which are toxic for the vascular endothelium and the lung parenchyma, responsible for a variable degree of respiratory distress–which may lead to ARDS. Symptoms typically occur within 24–96 hours after trauma. V/Q lung scan frequently demonstrates a typical mottled appearance with numerous subsegmental perfusion defects, even when chest X-ray is normal. Chest X-ray classically shows a “snow-storm” infiltration. Chest CT demonstrates nonspecific findings of permeability lung oedema, with bilateral areas of consolidation, often in a predominant dependent position. Early findings of small ill-defined and predominantly centro-lobular micronodules may represent small foci of alveolar oedema caused by ARDS or hemorrhage. Micronodules and areas of ground glass attenuation may predominate in the upper zones.

Neurologic symptoms are usually self-limited, although they may be definite in patients with poor outcome. Petechia, which strongly suggest FES, are seen in only 50% of patients, and usually after the onset of respiratory and neurologic symptoms. They are usually located in the nondependent areas of the head, neck and chest. Mortality for clinical FES is 5–20%.

## Other Embolism after Trauma

Various tissues may embolize into PAs after trauma. The presence of liver emboli has essentially medicolegal applications to determine if trauma is ante- or post-mortem, as it can be used as a sign of vital reaction. Cerebral emboli in the PAs are a direct and continuous source of coagulation disorder and recipients of transplanted lung from donors who underwent severe head trauma have been reported to present with early, severe and potentially lethal graft dysfunction due to multiple thrombi, diffuse alveolar damage and alveolar hemorrhage.

## Venous Air Embolism

The most common cause of venous air embolism (VAE) is central venous catheterization, particularly in conditions that decrease the intravenous pressure below ambient air pressure, such as strained respiration. VAE may also be directly associated with head, neck, thoracic or abdominal trauma or surgery. In the head and neck trauma, air enters the venous system due to frequent subatmospheric pressure in veins located above the heart. Pneumothorax, pneumomediastinum or bronchopulmonary-venous fistula can favour ingress of air in the systemic or pulmonary veins. Signs and symptoms vary from none to cardiovascular collapse, depending on the amount and rate of air entry and the position of the body. With a bolus administration, VAE may obstruct the right ventricular ejection. With infusion, the obstruction is located at the level of the pulmonary arterioles. In healthy humans, the fatal volume is estimated to be between 100 and 500 mL of air at 100 mL/sec. Signs of paradoxical emboli may be encountered in some patients by passage of air through a patent foramen ovale, across the pulmonary capillaries or arteriovenous pulmonary shunts. Chest X-ray is usually normal or shows pulmonary infiltrates reflecting pulmonary oedema. Most patients recover with supportive care including administration of 100% oxygen and/or hyperbaric oxygen therapy.

## Amniotic Fluid Embolism

Amniotic fluid embolism mainly follows an iatrogenic trauma that results in embolization of large amounts of amniotic fluid debris within PAs. The delay between trauma and clinical symptoms may vary from 0 to 48 hours. The cardinal signs associate cyanosis, cardiovascular collapse, sudden onset of respiratory distress, major coagulation disorder in 40% of the patients and signs of central nervous system irritability in 20%. CT may demonstrate signs of permeability oedema, ground glass opacification and centrilobular nodules. Massive PE due to amniotic embolism has also been reported. Maternal mortality is high, 80 to 100%, of which 25 – 50% die within one hour of symptoms onset. Diagnosis is usually performed by blood aspiration of amniotic fluid debris from the pulmonary circulation. The presence of fetal material in the pulmonary circulation can nevertheless be found in asymptomatic women, which suggests the presence of subclinical amniotic fluid embolism.

## Foreign Body Embolism

**Figure 3:**
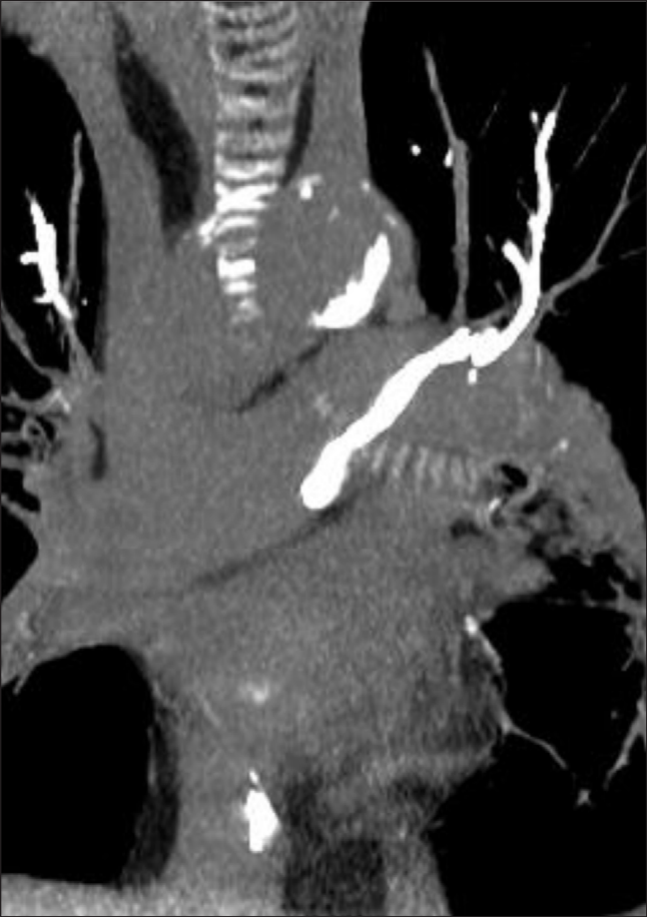
Embolization into the pulmonary arteries of acrylic cement leak after vertebroplasty.

Foreign body embolism into the PAs is increasingly seen in clinical routine. Most are iatrogenic and include among others fragments of catheter, vertebroplasty cement, radioactive seeds, lipiodol, coils and other sclerotherapy material (Figure 3). It may also be seen in IV drug abusers or after a suicide attempt with various substances including among others mercury, talc or cellulosis.

In conclusion, nearly any endogeneous and exogeneous material can embolize into PAs. Clinical and radiological presentations are variable and some may simulate acute pulmonary embolism. A prompt and correct diagnosis is required as the treatment is specific to each condition.
